# Microstructure Characterization and Mechanical Properties of Stainless Steel Clad Plate

**DOI:** 10.3390/ma12030509

**Published:** 2019-02-08

**Authors:** Hao Li, Liyuan Zhang, Boyang Zhang, Qingdong Zhang

**Affiliations:** School of Mechanical Engineering, University of Science and Technology Beijing, Beijing 100083, China; lihaoustb@163.com (H.L.); ustbzly@163.com (L.Z.); zbyustb@163.com (B.Z.)

**Keywords:** stainless steel clad plate, element diffusion, microstructure, mechanical properties

## Abstract

In this study the microstructure and mechanical properties of stainless steel clad plate are researched. Due to element diffusion (Fe, Cr, Ni, Mn), a 20 μm thick diffusion layer is formed between stainless steel and carbon steel clad plate. The diffusion layer has a stable mechanical performance without obvious grain microstructure, and its internal mechanical properties show a graded change in the thickness direction. This is beneficial to a strong bond between stainless steel and carbon steel and the stable transition of mechanical performance in the thickness direction, as well as further carbon diffusion changes in the microstructure and mechanical properties near the diffusion layer of clad plate. Carburization stainless steel with a thickness of 150 μm is formed in the stainless steel side and decarburization carbon steel with a thickness of 80 μm is formed in the carbon steel side.

## 1. Introduction

Metal clad plates with good properties and various functions have developed in many industries [1,2,3]. Among them, stainless steel clad plates are one of the most widely used products. Stainless steel clad plates not only have good weldability, ductility and thermal conductivity from carbon steel, but also hold high corrosion resistance, abrasion resistance and magnetic resistance from stainless steel [4,5]. Various technologies are utilized to produce metal clad plates, for instance, explosion welding [6], diffusion bonding and roll bonding [7,8].

The composite mechanism and interface microstructure of metal clad plate have been extensively researched. Previous studies suggest that element diffusion occurs near the interface of clad plate, which is possibly key to the bonding of metal clad plate [9,10,11]. For stainless steel clad plate, many studies report that there is an element diffusion layer between stainless steel and carbon steel [12,13]. Further studies show that carbon diffusion occurs between carbon steel and stainless steel clad plate, which results in the formation of a decarburization layer in the carbon steel side, and a carburization layer in the stainless steel side, respectively [14,15,16]. Compared to carbon steel and stainless steel, the decarburization layer and carburization layer have different microstructures and mechanical properties which could affect the mechanical performance of clad plate. The studies on the diffusion layer, decarburization layer and carburization layer in stainless steel clad plate have become increasingly active, but most studies remain in the observation stage [17,18], and only a few studies conducted some simple mechanical tests on them [19,20].

In this paper, the microstructure characterization of stainless steel clad plate will be researched and classified, and the main mechanical properties of the different microstructures in clad plate will be tested systematically. Investigating the microstructure and mechanical properties of clad plate can be a guide to deformation behavior research and finite element modeling of stainless steel clad plate.

## 2. Materials and Methods

The stainless steel clad plate was fabricated by hot-roll bonding. Q235 carbon steel was chosen as the substrate and 304 stainless steel was chosen as the cladding. The chemical compositions of the metals are listed in Table 1.

Before the rolling process, the surfaces of stainless steel and carbon steel were properly cleaned by a grinding machine (Au-1550, Zhongshuo, Shanghai, China) to remove contaminants or any oxide layer. Then carbon steel was stacked with stainless steel and the plate edges were welded with four carbon steel seals. A hole was drilled in the middle of one seal, and the air in the plate interface was pumped out through the hole by a vacuum pump (H-180, EVP, Shanghai, China) reaching a proper vacuum degree (0.5 Pa—5 Pa). After being heated to a temperature of 1100 °C for 3 h in a pit furnace (B1-1200, Grieve, Round Lake, USA), the plate was multiple-pass rolled at a temperature of 800 °C before being cooled in the air. The specimens cut from the clad plate were polished and cleaned. 

Due to the difference of corrosion potential between stainless steel and carbon steel, the respective clad plate formed a galvanic couple in the etchant. The galvanic couple provided anodic protection against the corrosion of stainless steel, which could affect its metallographic corrosion. In view of this, the metallographic corrosion of stainless steel and carbon steel was conducted separately. The carbon steel clad plate was etched with 4% nitric acid alcohol solution. The carbon steel clad plate was removed by a low-concentration nitric acid solution before the stainless steel clad plate was etched with a mixed aqueous solution of 4% nitric acid and 6% hydrofluoric acid for 20 min at room temperature. After metallographic corrosion, the microstructure and element distribution of the specimens in the thickness direction were observed by scanning electron microscopy (SEM, Sigma-300, Zeiss, Oberkochen, Germany) and electron probe micro analyzer (EPMA, EPMA-1720, Shimadzu, Kyoto, Japan), respectively.

**Figure 1:**
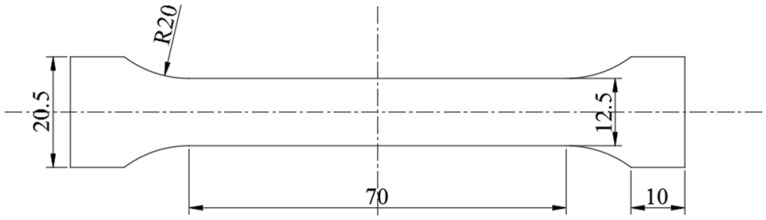
Schematic of the tensile sample (in mm).

To investigate the mechanical properties of stainless steel clad plate, tension tests were conducted. The tension specimens were fabricated by wire-cut electrical discharge machining (WEDM, MP1200, Mitsubishi Electric, Tokyo, Japan) under proper cooling and lubrication conditions. The WEDM has a displacement sensitivity of 0.005 mm and a precision of 0.003 mm, which is fit for metallic foil processing. Figure 1 shows the tensile sample prepared according to the ASTM E345-16 standard [21] and D638-14 standard [22], and the tensile sample thickness would be determined by the thickness of every parts of clad plate. The nano-hardness and elastic modulus distribution in the thickness direction of the clad plate were tested by a nano-indenter under an indentation depth of 0.003 mm for 20 s, and with indentation spacing of 0.005 mm to avoid any interaction between indentations.

## 3. Results and Conclusions

**Figure 2:**
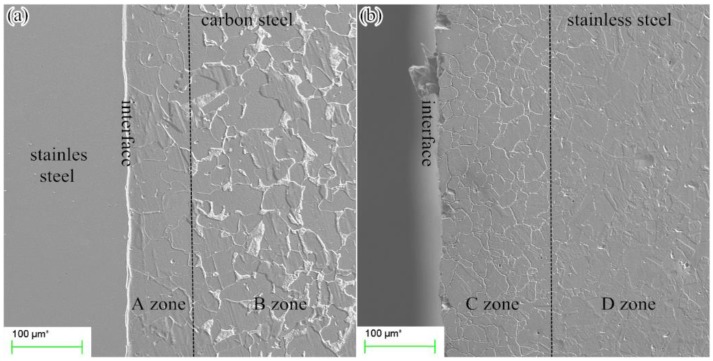
Microstructures of stainless steel clad plate (**a**) carbon steel side; (**b**) stainless steel side.

Figure 2a shows the microstructure of the carbon steel side of the clad plate. The carbon steel is etched while the stainless steel is not etched, indicating that stainless steel has a better corrosion resistance than carbon steel. The carbon steel further from the interface (B zone) is comprised of pearlite (the white structure) and ferrite, which are the general components of carbon steel. The carbon steel nearer the interface (A zone) with a thickness of 80 μm is mainly composed of ferrite. The pearlite in A zone cannot be observed obviously which indicates some loss. Figure 2b shows the microstructure of the stainless steel side of the clad plate. Based on the different grain boundaries and ASTM A262-15 standard [23], the stainless steel of the clad plate can be divided into two parts. In the part nearer the interface (C zone), all grains are surrounded by ditches. The C zone has a thickness of 150 μm. In the part further from the interface (D zone), there are only steps between grains and no ditches on the grain boundaries, which is the general composition of stainless steel.

**Figure 3:**
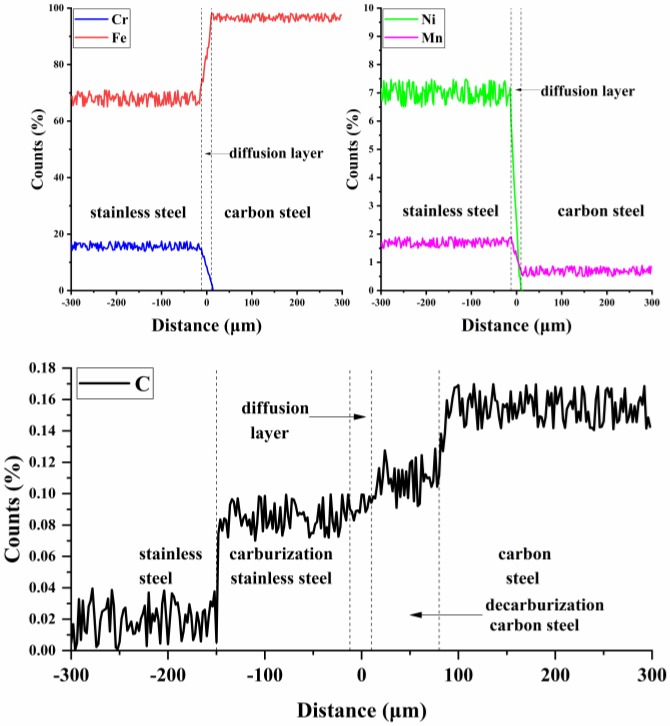
The EPMA line of stainless steel clad plate.

Figure 3 shows the major element distribution in the thickness direction of clad plate. The content of Fe, Cr, Ni and Mn are basically unchanged in the stainless steel and carbon steel sides. However, there is an element transition layer between stainless steel and carbon steel which has a thickness of 20 μm. In the layer of Cr, Ni and Mn, content decreases linearly from the stainless steel side to the carbon steel side whereas the content of Fe increases linearly. The gradient transfer of major elements indicates that the element diffusion has occurred between stainless steel and carbon steel, forming a diffusion layer. However, the diffusion layer cannot be distinguished obviously in Figure 2, possibly because the element diffusion does not change the grain morphology and the characteristic scale of the diffusion layer. In addition, A zone and C zone as shown in Figure 2 cannot be distinguished by the major element (Fe, Cr, Ni, Mn) distribution in the thickness direction of clad plate. However, the carbon distribution in the thickness direction of clad plate corresponds to the microstructure of clad plate in the thickness direction. The average carbon content of A zone, B zone, C zone and D zone as shown in Figure 2 are 0.12%, 0.16%, 0.08% and 0.03%, respectively. Compared to B zone, the carbon content of A zone decreases from 0.16% to 0.12%, which corresponds to the loss of pearlite in A zone, indicating the decrease of carbon content of A zone. When compared to D zone, the carbon content of C zone increases from 0.03% to 0.08%, and the increase of carbon content changes the grain boundary of stainless steel, forming a new microstructure in C zone as shown in Figure 2. The different carbon contents between A zone and C zone indicate that carbon diffuses from A zone to C zone. Based on their different carbon contents, the A zone and C zone can be called decarburization carbon steel and carburization stainless steel, respectively.

**Figure 4:**
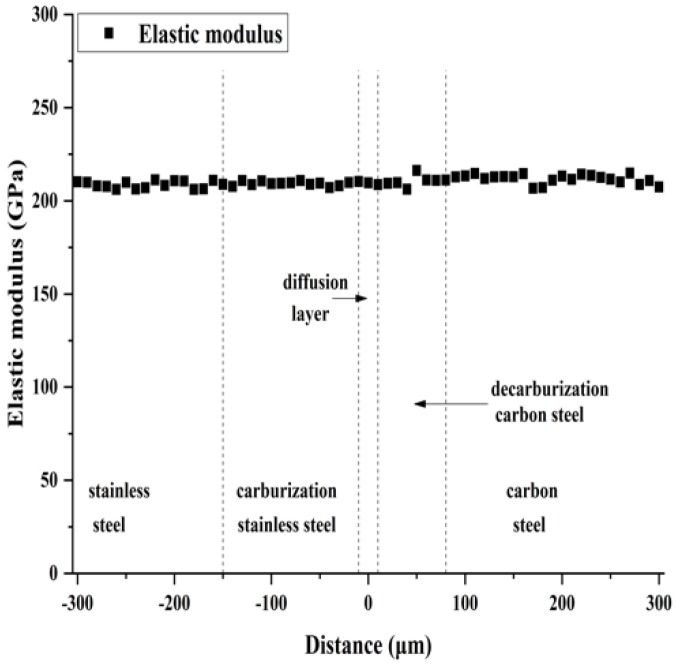
The elastic modulus distribution of stainless steel clad plate.

**Figure 5:**
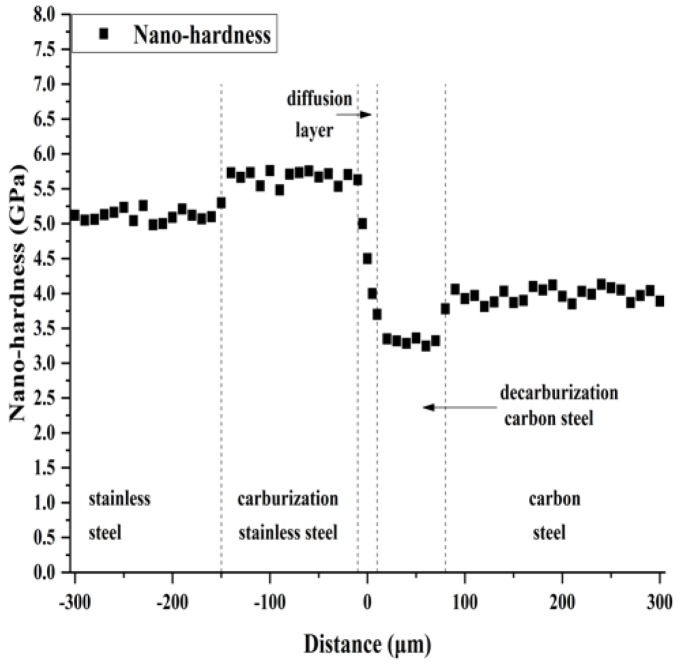
The nano-hardness distribution of stainless steel clad plate.

Figure 4 shows the elastic modulus distribution in the thickness direction of clad plate. The average elastic modulus of clad plate is 210 GPa, and the elastic modulus in the thickness direction does not show any marked changes in the different zones. Figure 5 shows the nano-hardness distribution in the thickness direction of clad plate. In the stainless steel side, the average nano-hardness of stainless steel and carburization stainless steel are 5.1 GPa and 5.7 GPa, respectively, which indicates that the carbon content increase improves the nano-hardness of carburization stainless steel. In the carbon steel side, the average nano-hardness of decarburization carbon steel and carbon steel are 3.3 GPa and 3.7 GPa, respectively. The nano-hardness decrease of decarburization carbon steel can be attributed to the decrease of carbon content, which is expressed by the loss of pearlite in decarburization carbon steel. In the diffusion layer, the nano-hardness decreases from carburization stainless steel side to the decarburization carbon steel side. Because the nano-hardness is linearly related to material plastic deformation [24,25], it can be said that the major mechanical properties of the diffusion layer show a graded change in the thickness direction. The nano-hardness achieves a stable transition from stainless steel to carbon steel through the diffusion layer, however carburization stainless steel and decarburization carbon steel enhance the nano-hardness difference between the stainless steel side and the carbon steel side.

**Figure 6:**
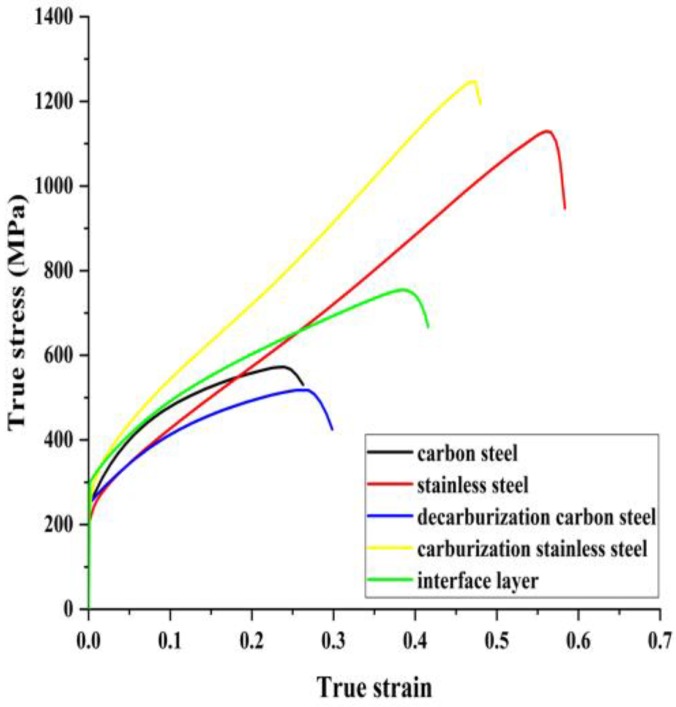
Stress–strain responses of different microstructures of stainless steel clad plate.

The tensile sample thickness of stainless steel, carburization stainless steel, the diffusion layer, decarburization carbon steel and carbon steel are 0.5 mm, 0.13 mm, 0.02 mm, 0.06 mm and 0.5 mm, respectively. And Figure 6 shows the stress-strain curve of each part of the clad plate, and the corresponding tensile properties are listed in Table 2. The yield strength of stainless steel, carburization stainless steel, the diffusion layer, decarburization carbon steel and carbon steel are 260 MPa, 310 MPa, 300 MPa, 240 MPa and 250 MPa, respectively. Compared to carbon steel, the decarburization carbon steel has a lower tensile strength (517 MPa) and higher ductility (29.8%). Compared to stainless steel, the carburization stainless steel has a higher tensile strength (1246 MPa) and lower ductility (47.5%). The whole diffusion layer has a stable tensile performance, which indicates that it can effectively transfer load and deformation as an interface bonding zone between stainless steel and carbon steel, avoiding the potential interface fracture.

From Figure 5 and Figure 6, and Table 2, it can be seen that carburization stainless steel and decarburization carbon steel increase the difference in mechanical properties between the stainless steel side and the carbon steel side, which could induce stress concentration near the interface during the clad plate deformation. However, due to the diffusion layer, the mechanical properties could achieve a stable transition in the thickness direction of clad plate, avoiding the stress concentration and potential interface fracture during the clad plate deformation.

## 4. Conclusions

Based on microstructure and element distribution, stainless steel clad plate in the thickness direction can be divided into five parts which are stainless steel, carburization stainless steel, diffusion layer, decarburization carbon steel and carbon steel. Due to the major element (Fe, Cr, Ni and Mn) diffusion between stainless steel and carbon steel, a 20 μm thick diffusion layer without obvious grain microstructure is formed. Due to further carbon diffusion from carbon steel to stainless steel, decarburization carbon steel with a thickness of 80 μm appears in the carbon steel side, having a higher ductility and a lower tensile strength than carbon steel. Further, carburization stainless steel with a thickness of 150 μm appears in the stainless steel side, having a lower ductility and a higher tensile strength than stainless steel. Through the diffusion layer, the main mechanical properties in the thickness direction of clad plate achieve a stable transition from the stainless steel side to the carbon steel side, which is beneficial to a strong bond between stainless steel and carbon steel.

## Figures and Tables

**Table 1:** Chemical composition of stainless steel and carbon steel (wt. %).

Elements	Cr	Ni	Mn	C	S	Si	P	Fe
SUS304	18	8	2	0.04	0.02	0.8	0.025	balance
Q235	0	0	1	0.17	0.04	0.3	0.045	balance

**Table 2:** Tensile properties of different microstructures of stainless steel clad plate.

Material	Yield Strength/MPa	Tensile Strength/MPa	Ductility/%
stainless steel	260	1129	58.3
carburization stainless steel	310	1246	47.5
diffusion layer	300	753	41.5
decarburization carbon steel	240	517	29.8
carbon steel	250	572	26.2
